# The Management and Prognostic Prediction of Adenocarcinoma of Appendix

**DOI:** 10.1038/srep39027

**Published:** 2016-12-16

**Authors:** Xin Xie, Zhangjian Zhou, Yongchun Song, Wenhan Li, Dongmei Diao, Chengxue Dang, Hao Zhang

**Affiliations:** 1 Department of Surgical Oncology, The First Affiliated Hospital of Xi’an Jiaotong University, 227W Yanta Road, Xi’an, 710061, Shaanxi, China

## Abstract

Malignant tumours of the appendix are quite rare, especially appendiceal adenocarcinomas, which may be difficult to detect preoperatively or intraoperatively. We collected data for 1404 patients with adenocarcinoma of the appendix from the Surveillance, Epidemiology, and End Results Program (SEER) database to explore the potential associations between clinicopathological factors and overall survival. Furthermore, a novel nomogram for predicting prognosis was developed based on our analysis of the SEER data. The nomogram prediction model included seven prognostic factors derived based on different clinical estimates. When compared with the traditional tumour-node-metastasis (TNM) staging system, the nomogram prediction model showed superior discriminatory power (Harrell’s C-index, 0.741 vs. 0.686) and a greater degree of similarity to actual 5-year overall survival after calibration (Akaike Information Criterion index, 5270.781 vs. 5430.141). Finally, we provide recommendations for the management of patients with adenocarcinoma of the appendix. Notably, we found the depth of adenocarcinoma invasion may be used as an indicator to determine the optimal surgical approach. For mucinous adenocarcinomas of the appendix, because these tumours are characterized by unique biological behaviour, intraoperative hyperthermic intraperitoneal chemotherapy (HIPEC) is recommended. However, whether systematic chemotherapy should be administered to patients with adenocarcinoma of the appendix requires further investigation.

Malignant neoplasms of the appendix are extremely rare, with an age-adjusted incidence that has been estimated to be approximately 0.12 per 1,000,000 person years^1^. According to the National Cancer Institute, based on data from the Surveillance, Epidemiology, and End Results (SEER) database, appendiceal cancer accounts for 0.4% of all gastrointestinal tumours^2^. Because carcinomas of the appendix may cause appendicitis or rupture of the appendix, the most common symptoms of primary appendix carcinomas present similarly to acute appendicitis. Therefore, appendiceal neoplasms are seldom detected before or during appendectomy, with less than 1.5% of the appendectomy specimens revealing primary appendiceal cancers upon examination^3^,^4^.

The appendix is embryologically derived from the colon, and while the function of appendix remains unclear, it has been proposed to play a role in immune function. The majority of appendiceal carcinomas are carcinoids (endocrine cell tumours), accounting for 85% of epithelial appendiceal tumours^5^; carcinoids often present with chronic recurrent right lower quadrant pain, which may be difficult to distinguish from acute appendicitis^6^. In addition, appendiceal carcinomas can be subdivided into mucinous adenocarcinomas, colonic-type adenocarcinomas, adenocarcinoids with dual cell origin and signet-ring cell adenocarcinomas^1^,^6^.

Adenocarcinomas of the appendix are primary malignant neoplasms of the appendix that comprise mucinous, non-mucinous (colonic-type), and signet-ring cell adenocarcinomas^1^. While the prognostic factors for mucinous adenocarcinomas and non-adenocarcinomas remain poorly understood, worse prognosis has been observed in patients with signet-ring cell adenocarcinomas.

Within the American Joint Committee on Cancer (AJCC) Staging Manual, 7^th^ edition, appendiceal carcinomas are listed as an independent category separate from colorectal carcinomas^7^. Moreover, mucinous adenocarcinomas of the appendix are categorized by histological grade into low-grade and high-grade (well-differentiated and moderately/poorly differentiated, respectively) tumours in the AJCC TNM Staging System.

Though well-differentiated adenocarcinomas of the appendix have been found to be associated with better prognosis than poorly differentiated adenocarcinomas, and histological grade, as defined by the AJCC TNM system, may serve as an important predictor of appendiceal adenocarcinoma patient prognosis, it has been noted that there was a significant difference in cancer-specific survival between patients with moderately and poorly differentiated adenocarcinomas^8^. In our study, we developed a novel prediction model that was based on data from the SEER database and may provide a better and more accurate prediction model for prognosis in appendiceal adenocarcinoma patients.

## Results

### Demographic and pathological characteristics of patients

A total of 1404 patients with adenocarcinoma of the appendix who were reported in the SEER database from 2004 to 2013 fulfilled the inclusion criteria (Table 1). Of these patients, 50.5% were male (n = 709), and the remaining 695 patients were female. The average age of patients was 61.3 ± 14.4 years, ranging from 20 to 101 years (median age of 66 years). Most of the patients were Caucasian, and 11% and 7.4% of patients were black and other ethnicities (including Chinese and Japanese descent), respectively. Approximately 36% of the patients were single (including never married and divorced patients) when they were diagnosed with appendiceal adenocarcinoma. Regarding pathological characteristics, 427 patients had distant metastasis identified when they underwent the operation. In approximately 18.4% of patients, the tumour had not invaded the serosa. In 36.9% of patients, the tumour was invading the serosa, and in the remaining 44.7% of patients, the adenocarcinoma had invaded the serosa. No regional metastatic lymph nodes were present in most of the patients, and more than half of the patients had at least 12 regional lymph nodes resected. Of the 1404 patients, 18.1% had adenocarcinoma of the appendix that was poorly differentiated or undifferentiated in histological grade. Approximately 70% of patients underwent extended resection (including hemicolectomy or total colectomy) of appendiceal adenocarcinoma. In addition, 30% of the tumours had a diameter of more than 50 millimetres.

### The overall survival in patients with appendiceal adenocarcinoma

In the multivariate analysis, we could found that patients who were less than 50 years old, married, and had well-differentiated adenocarcinoma, no serosal invasion, more than 12 resected lymph nodes with no metastasis and no distant metastasis had significantly better 5-year overall survival rate than their respective counterparts (Table 1). However, when we subdivided the patients into stage IV and stage I-III according to the AJCC Staging Manual, 7^th^ edition, we found that patients who had mucinous adenocarcinoma had significantly poor 5-year overall survival. Other risk factors identified in the two different groups are shown in Table 2.

### The nomogram prediction model for adenocarcinoma of the appendix

**Figure 1:**
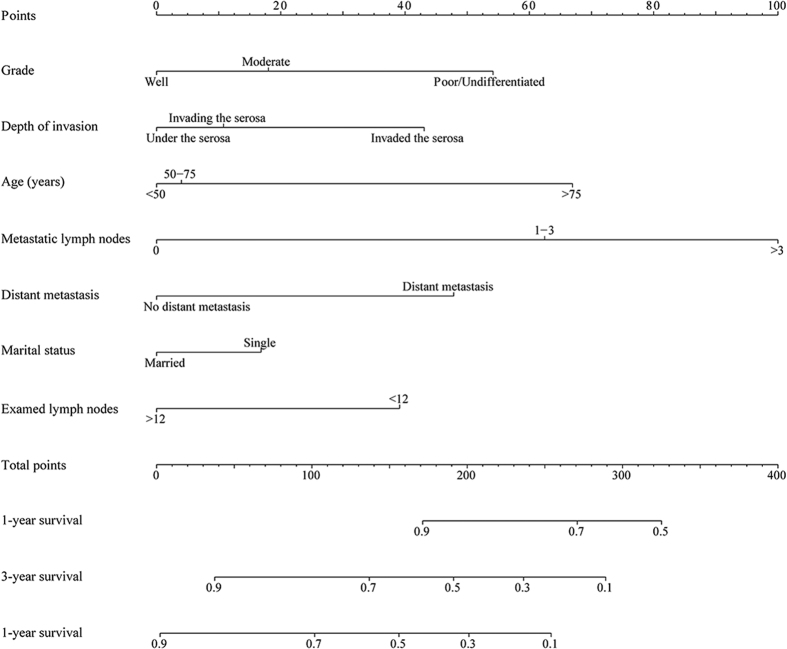
Nomogram predicted 1- to 5-year overall survival using six available clinical characteristics.

**Figure 2:**
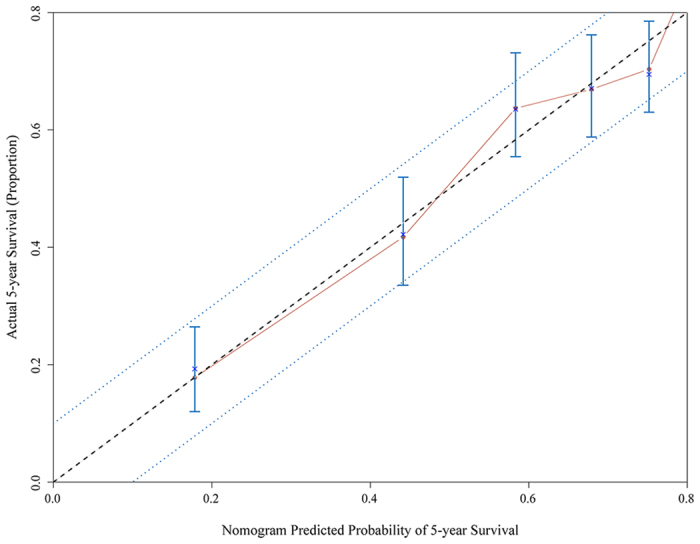
Calibration of the nomogram predicted system. Nomogram predicted probability of overall survival was plotted on the x-axis, actual overall survival was plotted on the y-axis and 95% CIs measured by Kaplan-Meier analysis. All predictions lie within the 10% margin of error (within the blue dots line).

For patients who underwent surgical treatment, seven prognostic factors, age at diagnosis the appendiceal adenocarcinoma, marital status, depth of tumour invasion, total number of resected regional lymph nodes, number of metastatic lymph nodes, histological grade and distant metastatic status of the adenocarcinomas, were included in the nomogram model. In the nomogram model, each factor from the multivariate Cox proportional hazard regression model was ascribed a weighted point that implied survival prognosis. For example, 60 years old was associated with 4 points, invasion of the serosa was associated with 43 points, 1 lymph node metastasis was associated with 62 points, 18 resected lymph nodes were associated with 0 points, moderately differentiated adenocarcinoma was associated with 18 points, and married status and the presence of no distant metastasis were associated with 0 points; therefore, a total of 127 points were possible. In addition, for each patient, a higher score was considered to predict worse prognosis. The final nomogram model used to predict the survival (1-year, 3-year, 5-year overall survival) of patients with adenocarcinoma of the appendix undergoing surgical resection is shown in Fig. 1. The predictive accuracies of the final nomogram model and the traditional AJCC TNM staging system were evaluated using the Harrell’s C-index and Akaike information criterion (AIC) index. For the nomogram model, the C-index was 0.741, which indicated that the model had better discriminatory ability than did the traditional AJCC TNM staging system, which had a C-index of 0.686. Figure 2 shows the calibration plot of the 5-year survival nomogram. As indicated in this figure, predicted survival corresponded closely with actual survival and was always within a 10% margin of error. To avoid overfitting the prognostic models, AIC indices were calculated. The AIC index of the nomogram model was 5270.781, which was lower than that of the traditional AJCC TNM staging system (AIC of 5430.141). This result indicated that the nomogram model was better at predicting prognosis than was the traditional system and did not overestimate the actual 5-year overall survival rate.

### The relationship between the nomogram model and AJCC TNM staging system

**Figure 3:**
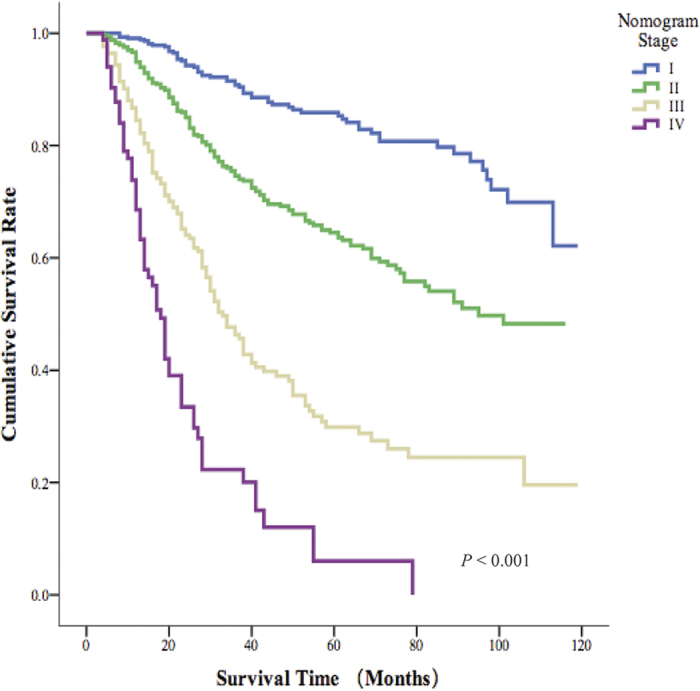
Kaplan-Meier survival curves of 4 nomogram stages of patients with appendix adenocarcinoma.

**Figure 4:**
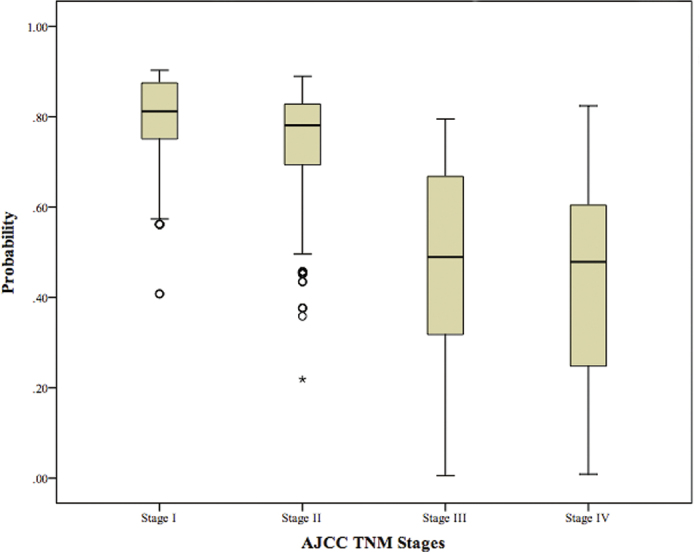
The overall survival probability distribution of different AJCC stages.

**Figure 5:**
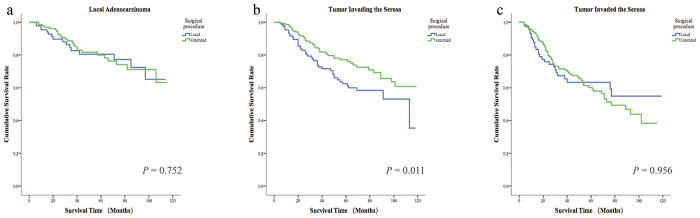
5-year overall survival of different surgical procedure according to different depth of tumor invasion. (**a**) For tumor did not invade the serosa, the 5-year overall survival had no significant difference of the two kinds of surgical approach. (**b**) For tumor was invading the serosa, the extended surgery had a significantly better 5-year overall survival. (**c**) For tumor had invaded the serosa, the 5-year overall survival had no significant difference of the two kinds of surgical approach.

**Figure 6:**
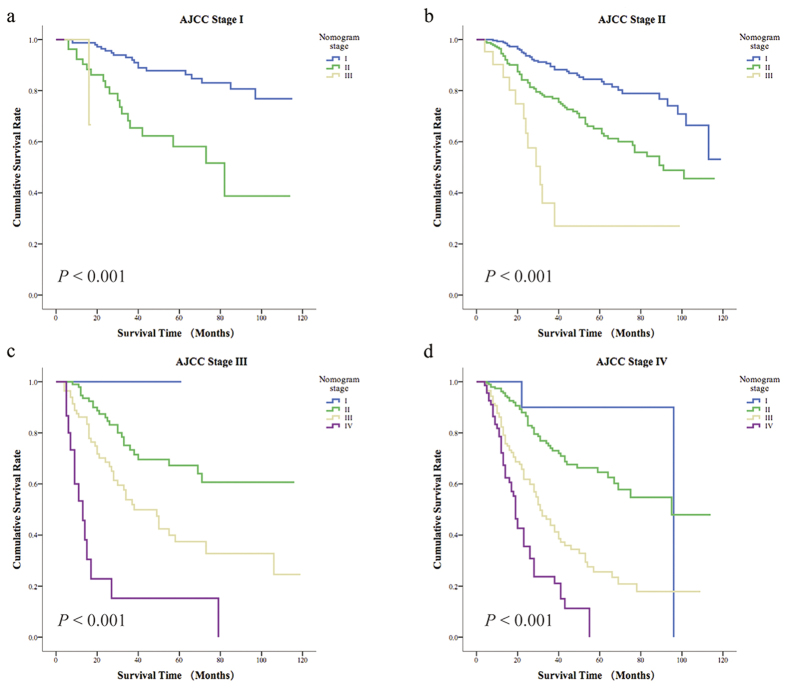
The 5-year overall survival of 4 nomogram stages of each AJCC TNM stage. (**a**) AJCC TNM stage I. (**b**) AJCC TNM stage II. (**c**) AJCC TNM stage III. (**d**) AJCC TNM stage IV.

Based on the AJCC TNM staging system, we subdivided the nomogram scores into four stages. As shown in Fig. 3, we found that the overall 5-year survival rates of patients in the four nomogram stages were significantly different. For the different AJCC TNM stages, patients in the same stage had different overall survival probabilities (Fig. 4). In addition, the overall survival rate of patients in different nomogram stages was significantly different from their survival rates predicted according to the different AJCC TNM stages (Fig. 5). However, when we compared the overall survival rates of patients in the four nomogram stages, we found that the survival rates of patients in the nomogram stages did not differ within each AJCC TNM stages (Fig. 6). Overall, as mentioned above, the nomogram prediction model was able to discriminate patients with appendiceal adenocarcinoma into survival risk groups in the grouped survival analysis with a high degree of homogeneity.

### Surgical approaches to adenocarcinomas of the appendix

**Figure 7:**
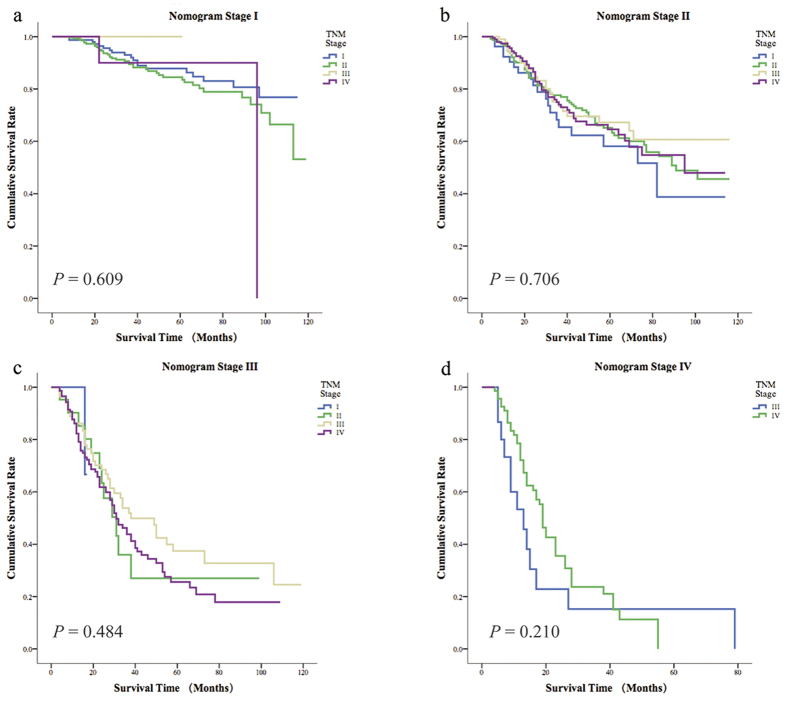
The 5-year overall survival of 4 AJCC TNM stages of each nomogram stage. (**a**) Nomogram stage I. (**b**) Nomogram stage II. (**c**) Nomogram stage III. (**d**) Nomogram stage IV.

The optimal surgical approach for patients with appendiceal adenocarcinoma remains controversial. More evidence is required to determine whether extended excision, such as hemicolectomy and total colectomy, or local excision should be considered as the best treatment option for patients. In our study, we found that in patients with adenocarcinomas localized in the mucosal layer, the overall 5-year survival rate did not differ between patients who underwent the two surgical procedures. However, when the tumour had penetrated the mucosal layer, patients who underwent extended resection had significantly better overall survival (Fig. 7).

## Discussion

Appendiceal cancer may be difficult to assess preoperatively and intraoperatively because of its rare incidence. The main types of appendiceal carcinomas are carcinoids, adenocarcinomas, adenocarcinoids, and signet-ring cell carcinomas. In this study, we analysed data for 1404 patients with appendiceal adenocarcinoma from the SEER database and demonstrated correlations between age, marital status, histological grade, TNM stage and overall 5-year survival rate in patients with appendiceal adenocarcinoma. Moreover, our analysis also showed that these factors, which may impact patient survival rate, were not independent. Notably, we found that patients with stage IV mucinous tumours, as indicated by the AJCC Staging Manual, 7^th^ edition, had poorer overall 5-year survival. Finally, for patients who underwent surgical therapy, we constructed a nomogram model to determine prognostic factors for survival. In comparison with the AJCC TNM staging system, the derived nomogram stages were better and more accurate at predicting survival in patients with appendiceal adenocarcinoma.

In accordance with previous studies, we identified several factors that could predict prognosis in patients with appendiceal adenocarcinoma. As mentioned above, younger age (<50 years old), lower TNM stage (no serosal invasion or distant metastasis), >12 resected lymph nodes without metastasis and well-differentiated histological grade were predictors of better overall 5-year survival. Interestingly, we also found that married status may serve as a protective factor, with survival in married patients exceeding that of single patients at a Hazard Ratio of approximately 0.78.

Mucinous adenocarcinomas are unique tumours of the appendix because of their particular biological behaviour. Compared with other types of appendiceal neoplasms, mucinous adenocarcinomas have greater potential to invade the serosa and spread to the peritoneum or abdominal cavity, which may result in pseudomyxoma peritonei (PMP). PMP is a rare clinical syndrome characterized by excessive accumulation of gel-like mucinous peritoneal fluid in the peritoneal or pelvic cavity, causing clinical symptoms such as abdominal pain, abdominal mass, progressive increases in abdominal circumference and weight loss^9^,^10^. The survival rate of patients with mucinous appendiceal adenocarcinoma is variable. While two previous studies reported better survival in these patients than patients with non-mucinous adenocarcinoma, one study reported worse prognosis in patients with mucinous than non-mucinous appendiceal adenocarcinoma, and another showed equivalent outcomes to be associated with both mucinous and colonic-type adenocarcinoma^1^,^5^,^11^,^12^. Michael J. Overman *et al*. found that mucinous adenocarcinomas were more likely to present with stage IV disease than were non-mucinous adenocarcinomas^8^. In our study, we subdivided the patients into stages I–III and stage IV, and our results showed that patients with mucinous adenocarcinoma had significantly poorer overall 5-year survival (Fig. 3), which suggests the presence of different biological behaviours in mucinous relative to non-mucinous adenocarcinomas.

While the molecular mechanisms of mucinous adenocarcinomas of the appendix remain poorly understood, previous studies have shown positive reactivity for CK20, CDX2 and MUC2 in these tumours^9^. In addition, K-ras mutations, p53 overexpression and microsatellite instability may also contribute to the development of mucinous tumours^8^,^13^.

In the 7^th^ edition of the AJCC Staging Manual, appendiceal carcinomas are classified separately from colorectal carcinomas^7^. However, while a recent study based on data from 2469 patients with appendiceal adenocarcinoma showed that markedly different outcomes were associated with mucinous and non-mucinous adenocarcinomas, these two subtypes of adenocarcinomas of the appendix are classified together in 7^th^ edition of the AJCC Cancer Staging Manual. Consistent with that study, we also identified stage IV mucinous adenocarcinomas of the appendix to be associated with significantly poorer overall 5-year survival than non-mucinous adenocarcinomas. Therefore, to predict the prognosis of patients with adenocarcinoma of the appendix more accurately, we need to explore the use of a better prediction system.

In our study, we developed a novel prognosis prediction system for patients with adenocarcinoma of the appendix, and specifically a nomogram prediction model. Based on the analysis of data from 1404 patients who underwent surgical treatment, we utilized seven factors to predict prognosis in patients with adenocarcinoma in the nomogram model. Each factor included in the nomogram model was ascribed a weighted point to estimate the effect of this factor on prognosis. In the nomogram model, a higher score indicated worse prognosis. Then, we appraised the predictive accuracy and homogeneity of the model relative to that of the traditional AJCC TNM staging system by calculating the Harrell’s C-index and Akaike information criterion (AIC) index, and the results suggested that the results of the nomogram prediction model corresponded more closely with actual survival (Fig. 2) and showed a lower AIC index, which meant that the nomogram model was a better prognosis prediction system (AIC index = 5270.81 of the nomogram model vs. 5430.141 of the AJCC TNM system). Furthermore, we demonstrated that the nomogram model produced results that were more homogenous than did the AJCC TNM stages. In brief, the nomogram prediction model appeared to be the preferred methodology to predict prognosis in patients with adenocarcinoma of the appendix.

Surgical procedures are important in the treatment of cancers, including appendiceal adenocarcinoma. However, explicit surgical guidelines for patients with appendiceal adenocarcinoma are not available. Whether local excision, hemicolectomy or total colectomy constitutes the best treatment option remains controversial. According to the study conducted by Kelly, for early-stage tumours of all subtypes of appendiceal cancer (including colonic-type adenocarcinoma, mucinous adenocarcinoma, goblet cell adenocarcinoma and neuroendocrine carcinoma) except goblet cell adenocarcinoma, appendectomy alone is recommended. For goblet cell adenocarcinoma, locally advanced adenocarcinoma or neuroendocrine carcinoma, however, right hemicolectomy, cytoreductive surgery followed with intraperitoneal chemotherapy (IPC) or systemic chemotherapy have been suggested as better treatment options^14^. Based on the analysis performed in our study, we found that depth of adenocarcinoma invasion could be used as indicator to determine the most appropriate surgical option. When the tumour was localized in mucosal layer, overall 5-year survival rates did not differ between patients who underwent local excision (*P = *0.752), such as appendectomy, and extended excision, such as hemicolectomy or total colectomy. If the tumour had invaded the mucosal layer, patients who underwent extended excision were found to have improved overall survival comparing relative to who only underwent localized resection (*P = *0.011 for tumour invading the serosa, and *P = *0.956 for tumour invaded the serosa). Notably, in our study, we identified no significant difference between the “tumour invaded serosa” group and the “tumour invading serosa” group. We hypothesize that this contradictory result might have resulted from the administration of postoperative systemic chemotherapy and limited duration of follow-up. Briefly, since the number of metastatic lymph nodes was identified as a significant prognostic factor, we preferred the use of right hemicolectomy for patients with tumours higher than the T2 stage.

Because peritoneal invasion in patients with adenocarcinoma of the appendix corresponds to stage IV in the AJCC TNM staging system, chemotherapy is usually used as an appropriate treatment for these patients. Mucinous adenocarcinomas of the appendix, because of their unique biology behaviour, may be more likely to be associated with the development of PMP if tumour cells spread into the peritoneal cavity. Thus, IPC is required for the treatment of mucinous adenocarcinoma and intraoperative hyperthermic intraperitoneal chemotherapy (HIPEC) is usually utilized. According to the recent consensus guidelines from the American Society of Peritoneal Surface Malignancy (ASPSM), intraoperative HIPEC using a closed method is recommended for colorectal cancer patients with peritoneal invasion^15^.

It should be noted that multidisciplinary therapies, especially surgical resection followed by adjuvant chemoradiation therapy, have been increasingly applied for the treatment of suitable patients with resectable digestive tract cancers^16^,^17^,^18^,^19^. However, whether multidisciplinary therapies could improve overall survival in patients with adenocarcinoma of the appendix remains uncertain. As mentioned above, mucinous adenocarcinomas of the appendix could be associated with the development of PMP, which requires cytoreductive surgery and HIPEC as first-line therapies^15^. In a recent study, Asare *et al*. demonstrated that systematic chemotherapy may have a significantly beneficial on overall survival regardless of adenocarcinoma histology (HR,0.79; 95% CI, 0.69–0.90; *P = *0.0005 for mucinous; and HR, 0.84; 95% CI, 0.75–0.95; *P = *0.004 for nonmucinous). However, in stage IV patients, the benefit of systematic therapy has been reported to be influenced by tumour grade and histology. In addition, mucinous and well-differentiated adenocarcinomas did not appear to benefit from systemic chemotherapy^20^. In our study, due to the limitations of the SEER database, we could not obtain information regarding the administration of chemotherapy in patients with adenocarcinoma of the appendix. This lack of information may be a potential limitation of our nomogram prediction model. However, based on a large cohort of patients with adenocarcinoma of the appendix reported over the course of ten years in the SEER database, more accurate predictions and more homogenous results were generated based on our nomogram model relative to the ATCC TNM system, and we believe our nomogram prediction model could serve as an accurate model for the prediction of prognosis in patients with adenocarcinoma of appendix in the future.

As cancer is a complex disease, high-quality prognostic biomarkers or models will be useful clinically to estimate proper therapeutic strategies or to predict prognosis of patients with neoplasms. Referring to the nomogram predict model, we have noted that different stages meant disparate therapies and discrepant prognosis between low- and high-risk patients, especially in stage I-III and stage IV adenocarcinoma of appendix. Furthermore, in some cancer types, biomarkers or models for prognosis prediction needs to be more stage-specific. For example, whether adjuvant therapy should apply to the patients with stage II colorectal cancer (CRC) remains controversial for past few decades^21^,^22^,^23^. In a recent study conducted by Gao *et al*. the combinatory cancer hallmark-based gene signature sets (CSS sets), a newly biomarkers, was identified to predict the prognosis and to estimate the adjuvant chemotherapy benefits of patients with stage II CRC accurately. It demonstrated that patients with high-risk stage II of CRC defined by the CSS sets gained significant survival benefits from adjuvant chemotherapy based on fluorouracil. However, on the contrary, there were not obviously survival benefits for the patients with low-risk and intermediate-risk stage II CRC^24^. In this study, as discussed above, different scores defined by our nomogram model are used to predict prognosis for patients with various stages in AJCC TNM system. Meanwhile, proper therapeutic strategies, such as adjuvant chemotherapy or HIPEC, are recommended for the patients with adenocarcinoma of appendix referring to nomogram model scores. However, because of the distinctive clinical symptoms of adenocarcinoma of appendix, pre-operative estimation is rare and limited. Therefore, estimation methods and multidisciplinary therapeutic strategies of adenocarcinoma of appendix need further exploration.

Based on the data obtained from the SEER database, there are both strengths and limitations to our study. Some clinical details that might influence the analysis of survival estimates are not included in the SEER database. For example, whether the patients underwent systematic chemotherapy or intraoperative IPC was not included. These missing data may be important, as IPC is a necessary therapy for patients with mucinous adenocarcinomas of the appendix and PMP syndrome. In addition, the administration of systematic chemotherapy may influence prognosis in cancer patients. Moreover, the classification of histological grade might have caused potential bias because of its subjective diagnostic criterion. Despite these two limitations, our study was based on a large sample size of approximately 1500 patients reported in the SEERS database over the course of 10 years, which may have diminished any potential biases in analysis. Furthermore, the application of stratified adjusted survival analysis may have generated more accurate results when exploring the relationships between potential prognostic factors and overall 5-year survival rates.

In conclusion, our novel nomogram prognosis prediction model, which contained seven clinical factors, generated estimates that were more accurate and homogeneous than those generated using the traditional AJCC TNM Staging system. This nomogram prediction model might help clinicians to predict survival in individual patients and select proper therapeutic strategies.

## Patients and Methods

### Patients

Data collected included the demographic and pathological characteristics and survival of patients with adenocarcinoma of the appendix. All patients were reported between 2004 and 2013 in the SEER database. The inclusion criteria were as follows: 1. Patients with pathologically diagnosed adenocarcinoma of the appendix; 2. Patients who underwent surgery and for whom exact pathological details were available; 3. Patients who survived for more than three months after surgery. In our study, a signed SEER research data agreement form was provided to the SEER Program and we were approved to access and analyze the SEER data. Because all data was collected from the SEER database, it did not require informed consent. This study was also approved by the ethical committee of the First Affiliated Hospital of Xi’an Jiaotong University.

### Statistical analysis

Continuous data are presented as means ± standard deviations. Categorical variables were grouped and compared using the χ^2^ test or Fisher’s exact test. Continuous variables were compared using the Student’s t-test. Univariate and multivariate Cox proportional hazard regression models were constructed to explore the associations between clinicopathological factors and overall survival. All parameters that were statistically significant in the univariate analysis were included in the multivariate Cox model. Overall survival was estimated using the Kaplan-Meier method, and differences in survival were examined using the log-rank test. Preselected multiple potential interactions were tested as nomogram parameters irrespective of significance. Akaike’s Information Criterion (AIC) and Harrell’s C statistic were used to estimate the accuracies and relative discriminatory abilities of the predictions. All statistical tests were two-sided, and *P* values < 0.05 were considered to be statistically significant. Statistical analyses were performed using SPSS 13.0 and R software version 3.3.0 (http://www.r-project.org) with the “SEERaBomb”, “rms” and “AICcmodavg” packages.

## Additional Information

**How to cite this article**: Xie, X. *et al*. The Management and Prognostic Prediction of Adenocarcinoma of Appendix. *Sci. Rep.* **6**, 39027; doi: 10.1038/srep39027 (2016).

**Publisher's note:** Springer Nature remains neutral with regard to jurisdictional claims in published maps and institutional affiliations.

## Figures and Tables

**Table 1:** The univariate and multivariate analysis of adenocarcinoma of appendix.

	N	Percentage (%)	Univariate		Multivariate	
			5-year Overall Survival	*P* value	*P* value	Hazard Ratio
Age						
Mean(SD)	61.3 ± 14.4					
Median(Range)	62 (20–101)					
Less than 50	300	21.4	65%			
50–75	838	59.7	68%			
More than 75	266	18.9	51%	<0.001	<0.001	1.304–1.795
Gender						
Male	709	50.5	65%			
Female	695	49.5	64%	0.572		
Race						
White	1145	81.6	65%			
Black	155	11	59%			
Other	104	7.4	65%	0.498		
Marital status						
Single	505	36	62%			
Married	899	64	66%	0.027	0.008	0.628–0.934
T Stage						
Tis/T1/T2	259	18.4	80%			
T3	518	36.9	70%			
T4	627	44.7	54%	<0.001	<0.001	1.167–1.615
Examined lymph nodes						
Less than 12	635	45.2	60%			
More than 12	769	54.8	69%	<0.001	<0.001	0.497–0.750
Lymph nodes status						
N0	1046	74.5	73%			
N1	211	15	50%			
N2	147	10.5	30%	<0.001	<0.001	1.645–2.155
Grade						
G1	405	28.8	73%			
G2	745	53.1	66%			
G3/G4	254	18.1	45%	<0.001	<0.001	1.168–1.531
Distance metastisis						
M0	977	69.6	72%			
M1	427	30.4	48%	<0.001	<0.001	1.188–1.600
Tumor size						
<20	390	27.8	71%			
20–50	609	43.4	63%			
50–80	280	19.9	59%			
>80	125	8.9	62%	0.009	0.799	0.883–1.100
Resection						
Local excision	472	33.6	63%			
Hemicolectomy	836	59.6	67%			
Total colectomy	96	6.8	52%	0.002	0.676	0.890–1.079
Mucinous						
Positive	684	47.8	65%			
Negative	720	51.3	64%	0.918		

**Table 2:** The univariate and multivariate analysis of adenocarcinoma of appendix according to AJCC TNM staging system.

	Stage I–III				Stage IV			
	N	Univariate	Multivariate		N	Univariate	Multivariate	
		*P* value	*P* value	Hazard Ratio		*P* value	*P* value	Hazard Ratio
Age								
Less than 50	179				121			
50–75	580				258			
More than 75	218	<0.001	<0.001	1.531–2.326	48	0.633		
Gender								
Male	551				158			
Female	426	0.815			269	0.246		
Race								
White	792				353			
Black	120				35			
Other	65	0.895			39	0.063		
Marital status								
Single	350				115			
Married	627	0.002	0.001	0.505–0.849	272	0.688		
T Stage								
Tis/T1/T2	235				24			
T3	441				77			
T4	301	<0.001	<0.001	1.219–1.773	326	0.116		
Examined lymph nodes								
Less than 12	399				236			
More than 12	578	<0.001	<0.001	0.459–0.775	191	0.554		
Lymph nodes status								
N0	773				273			
N1	137				74			
N2	67	<0.001	<0.001	1.467–2.169	80	<0.001	<0.001	1.423–2.067
Grade								
G1	265				140			
G2	565				180			
G3/G4	147	0.002	0.043	1.007–1.536	107	<0.001	<0.001	1.303–2.021
Tumor size								
<20	319				71			
20–50	439				170			
50–80	168				112			
>80	51	0.481			74	0.245		
Resection								
local excision	336				136			
Hemicolectomy	612				224			
Total colectomy	29	0.086			67	0.934		
Mucinous								
Positive	382				302			
Negative	595	0.365			125	0.001	0.747	0.682–1.316
